# Mechanical loading induces primary cilia disassembly in tendon cells via TGFβ and HDAC6

**DOI:** 10.1038/s41598-018-29502-7

**Published:** 2018-07-23

**Authors:** Daniel T. Rowson, Julia C. Shelton, Hazel R. C. Screen, Martin M. Knight

**Affiliations:** Institute of Bioengineering and School of Engineering and Materials Science, Queen Mary University of London, London, United Kingdom

## Abstract

This study used isolated human tenocytes to test the hypothesis that cyclic mechanical strain directly stimulates primary cilia disassembly, and to elucidate the mechanisms involved. Cells were seeded onto flexible membranes and strained at 0–3%; 1 Hz, for up to 24 hours. Cilia length and prevalence progressively reduced with increasing strain duration but showed full recovery within 2 hours of strain removal. The response to loading was not influenced by actin organisation as seen in other cell types. However, the loading response could be recreated by treatment with TGFβ. Furthermore, treatment with the HDAC6 inhibitor Tubacin, or a TGFβ receptor inhibitor both prevented strain induced cilia disassembly. These data are the first to describe primary cilia expression in isolated tenocytes, showing that mechanical strain regulates cilia expression independent of changes in tendon extracellular matrix. Furthermore, we show that cilia disassembly is mediated by the activation of TGFβ receptors leading to activation of HDAC6. Previous studies have shown that cilia are required for TGFβ signalling and that tendon mechanosignalling is mediated by TGFβ. The present study therefore suggests a novel feedback mechanism whereby cilia disassembly inhibits prolonged TGFβ activation in response to continuous cyclic loading.

## Introduction

Tendons perform the primary function of transferring force from muscle to bone. As a result, these specialised collagenous tissues experience dynamic tensile mechanical loading and constant pre-stress due to muscle attachment. Tendons are easily injured and chronic tendon disease, known as tendinopathy, is both prevalent and poorly understood. The cells within tendon, have long been known to remodel the extracellular matrix (ECM) in response to changing loading conditions. This cellular response to mechanical loading is critical for tendon health and homeostasis and the retention of biomechanical functionality^1^.

Recent studies have shown that tendon cells exist within two distinct regions within the tendon, namely the collagen rich fascicular matrix (FM) and the surrounding proteoglycan rich interfascicular matrix (IFM). These regions have significantly different mechanical properties and experience different local mechanical loading conditions during normal activity^2,3^. Across tendon regions, both too much loading as well as too little appear to be harmful, and both excessive cyclic loading or stress deprivation have been shown to lead to catabolism and degeneration of the tissue^4–6^. In light of this, numerous studies have investigated the effect of mechanical loading, on tendon structure and cell metabolism, demonstrating changes in the expression of genes regulating matrix synthesis and catabolism^5,7^. In particular, studies have shown that cyclic loading causes up-regulation of gene expression for collagen I and matrix metalloproteases MMP1 and MMP3, via activation of the TGFβ signalling pathway^7^. TGFβ is a cytokine which regulates a number of vital cellular processes including proliferation, differentiation, growth and apoptosis. Interestingly, the application of TGFβ to tendon explants has been shown to mimic the effect of loading, leading to increased production of collagen^8^. TGFβ has also been shown to bind to and regulate proteoglycan synthesis in tendon^9^.

Primary cilia are slender, non-motile cellular organelles composed of an array of nine microtubule doublets which form an axoneme enclosed by a specialized cell membrane and projecting out from a ciliary pocket^10,11^. Their primary function is thought to be as a hub for various signalling pathways such as Wingless (Wnt) and Hedgehog (Hh) signalling as well as mechanosignalling^12–14^. More recent studies have also shown cilia involvement in growth factor signalling^15^, differentiation^16^, and inflammation^17^. The length of cilia, the proteomal content and hence the signalling functions are tightly controlled by the process of intraflagellar transport (IFT) which shuttles proteins on and off the axoneme.

Primary cilia are expressed singularly by almost every eukaryotic cell type in the human body^11^. In tendon, primary cilia have been measured at 1–2 μm in length^18,19^ and orientated in the direction of collagen fibres^20^. Our previous work found that tenocytes in the FM had primary cilia orientated in the direction of the collagen fibres and the applied tensile loading. However, tenocytes in the IFM had a more random orientation, reflecting the collagen organisation in this region^21^.

Primary cilia have been shown to be affected by mechanical load in a number of cell types. In articular chondrocyte primary cilia length is reduced by compressive^22^ or tensile loading^23^. In bone cells and epithelial cells, fluid shear forces also induce cilia disassembly and shortening^24^. It has been shown using tendon explants that cilia elongate under stress deprivation and that this can be reversed by cyclic loading^18^. Our previous work has shown that stress deprivation induces greater elongation in the IFM than the FM and that this correlates with larger mechanical degradation in the IFM^21^.

The few previous studies concerning tendon primary cilia response to loading have only examined static loading, unloading or low level cyclic strain. Therefore, little is known about the effect of high levels of cyclic tensile strain on tendon cell cilia. Similarly, the mechanism by which primary cilia length is regulated by mechanical loading of tendon cells is also unknown. Finally, no studies have investigated cilia length and prevalence in isolated tenocytes, which may provide a valuable model system for investigating tenocyte mechanosignalling.

While the exact mechanism for mechanical regulation of cilia length in tendon is unknown, there are a number of mechanisms known to be involved in cilia length changes in other cell types which may play a role^24^. Increasing actin tension is known to inhibit cilia elongation and it has been suggested that actin tension can be regulated by changes in loading^25–27^. The interaction of cyclic strain, cilia length and actin tension can be investigated using blebbistatin, which inhibits myosin and leads to reduced actin tension. A second potential mechanism involves TGFβ_1_. It has been shown that TGFβ_1_ which is released by loading, leads to cilia shortening and disassembly in osteoblasts^28^. Other studies have also shown that the tubulin deacetylase HDAC6 also regulates cilia elongation, associated with modulation of tubulin acetylation and polymerisation^28,29^. Furthermore, TGFβ receptors localise to the primary cilium and primary cilia are required for TGFβ signalling^15^.

In the present study we test the hypothesis that mechanical loading regulates tenocytes primary cilia length via a TGFβ dependent mechanism involving regulation of HDAC6 and actin tension. We show, for the first time, that cyclic loading of isolated tenocytes leads to dramatic primary cilia disassembly and shortening, and that this is dependent on activation of TGFβ receptors and HDAC6 but not associated with changes in actin organisation. We also show that similar cilia disassembly in response to cyclic strain occurs *in situ* in the fasicular matrix (FM) region of tendon fascicles but not in the IFM. As TGFβ signalling is known to be mediated by primary cilia and induced by tendon mechanical loading^7,15,28^, we suggest that mechanically induced cilia disassembly may represent a novel feedback mechanism, controlling TGFβ signalling and downstream response to mechanical loading. This pathway may provide new therapeutic targets for controlling tendon response to mechanical loading and the development of tendinopathy.

## Materials and Methods

### Tendon and tenocyte culture

Cell culture media consisted of Dulbeco’s Modified Eagles Medium (DMEM) with glutaMax (Thermofisher, Waltham, MA) and addition of penicillin (96 μg/ml) and streptomycin (96 μg/ml). Supplemented DMEM was used either with or without 10% foetal calf serum (Thermofisher, Waltham, MA). Serum was withheld from 24 hrs prior to cyclic loading to ensure cells are quiescent such that cilia length and prevalence are not influenced by cell proliferation. For experiments involving intact tendon tissue, rat tail tendon fascicles were dissected from 4 rat tails within 24 hrs of death, following a procedure to ensure a layer of IFM is maintained on each fascicle as described previously^21^. This tissue was obtained as waste from unrelated experiments. All isolated cell experiments were performed using human tenocytes obtained by digestion from healthy male semitendinosus tendons (n = 3, age 48 ± 3) obtained with full ethics approval and informed consent from all donors (Collaboration with Graham Riley, University of East Anglia, NRES Committee East of England – Essex, REC number 09/H0302/3). Isolated tendon cells were cultured in media with serum in flasks up to passage 2–4 (P2-4) prior to use in loading experiments. All experiments were carried out in accordance with QMUL experimental procedures and any relevant guidelines and regulations.

For initial analysis of cilia expression in isolated tenocytes, cells at P2 were seeded onto glass coverslips and cultured until confluence. Media was changed to serum free DMEM 24 hrs prior to fixation.

### Mechanical loading of isolated tenocytes and tendon fascicles

Uniform, uniaxial cyclic tensile strain (CTS) was applied to the isolated tenocytes using the Flexcell FX4000-T system with arctangle loading posts (Dunn Lab, Germany). For mechanical loading experiments, cells were cultured in Flexcell 6-well loading plates. The deformable elastic membranes at the base of each well were pre-coated by the manufacturer with collagen type I. In each well 50,000 cells were seeded onto the membrane and then cultured for 3 days in media with serum, before moving to serum free media for subsequent loading. All mechanical loading experiments were performed in serum free DMEM. Cells were subjected to uniaxial 0–3% CTS for 0, 5, 8 and 24 hrs at 1 Hz in a similar protocol to that used in a previous study^7^. A further group of cells were left to recover for 2 hrs after the application of 24 hrs strain. Unstrained controls were cultured in an identical manner but without the application of strain.

To determine the mechanism(s) involved in mechanoregulation of cilia expression, isolated cells (both with and without cyclic strain) were treated with myosin inhibitor Blebbistatin (10 μM), HDAC6 inhibitor Tubacin (1 μM), TGFβ_1_ (10 ng/ml) or the TGFβ-RI inhibitor SB 431542 hydrate (TGFβ-RI) (10 μM). All antagonists and agonists were prepared in DMEM without serum. All reagents unless otherwise stated were purchased from Sigma (Sigma-Aldrich, St Louis, MO).

Cells from up to 3 donors were used for every experiment, and three technical repeats (3 Flexcell wells) prepared for each condition.

Mechanical loading of intact rat tail tendon fascicles was conducted immediately following dissection using the Bose Electroforce cyclic tensile system (TA Instruments, USA). Loaded fascicles were housed in custom made chambers^30^ and subjected to 4% CTS for 24 hrs at 1 Hz in serum free media. Separate fascicles were maintained either in chambers at 4% static strain, or were left stress deprived in wells for 24 hours, all in serum free media as described in our previous paper^21^. Stress deprived and statically strained fascicles provided control conditions alongside freshly dissected fascicles. 4% strain was used to be consistent with our previous study, as well as to try and ensure that the cells were not under-loaded since cells experience lower strains than those applied to the fascicle at a gross level^31^. 6 fascicles were used per condition, all from a single tail. 6 fascicles from each of 3 rats were also dissected and used immediately for comparison with isolated cells.

### Immunofluorescence labelling

Visualisation of the cilia axoneme in isolated tenocytes and intact tendon fascicles was performed by immunolabelling of acetylated α-tubulin and/or the cilia membrane protein, ARL13b.

For isolated cells the collagen coated Flexcell membranes were cut from the well plates and cells were fixed with 4% paraformaldehyde (PFA) (5 mins, room temperature) and incubated overnight at 4 °C in phosphate buffered saline (PBS) with rabbit anti-arl13b (1:1000, Abcam, Cambridge, UK) and mouse anti-acetylated α-tubulin (1:1000, Sigma-Aldrich, St Louis, MO). Samples were washed with PBS containing 0.1% bovine serum albumin (BSA) and incubated at room temperature in corresponding 488 nm and 543 nm Alexa secondary antibodies (1 hr, 1:1000, Molecular Probes, Invitrogen, Eugene, OR). To investigate the expression of F-actin, an additional 3 wells of cells were prepared for both the strained and unstrained conditions and were stained with phalloidin-Alexa488 (Thermofisher, Waltham, MA). The Flexcell membranes were finally placed under glass before imaging.

For visualisation of cilia in intact tendon, the fascicles were fixed in 100% methanol (2 hrs, room temperature) before washing in PBS with 0.1% BSA. Fascicles were incubated overnight at 4 °C with rabbit anti-arl13b (1:100, Abcam, Cambridge, UK) to stain the cilia axoneme, after which they were washed and incubated at room temperature with a 488 nm Alexa secondary antibody (1 hr, 1:1000, Molecular Probes, Invitrogen, Eugene, OR).

In both isolated cells and intact tendon fascicles, the nuclei were counter stained by incubation at room temperature with DAPI (5 mins, 1:5000, Molecular Probes, Invitrogen). After a further wash in PBS (5 mins) cells or fascicles were mounted under glass using prolong Diamond mountant.

### Confocal microscopy and analysis of primary cilia expression

All imaging was done using a 63×/NA 1.4 objective with a Zeiss laser scanning confocal microscope (Elyra, Zeiss, Oberkochen, Germany). Confocal z series were captured with a format of 225 × 225 μm and a pixel size of 0.11 × 0.11 μm and a z-step size of 0.25 μm. For isolated cells, between 20 and 30 sections were used per stack covering the full depth of cells on the slide. For intact fascicles between 20 and 30 sections were taken through the IFM and a further 20 to 30 through the FM as described in our previous paper^21^. Following 3D reconstruction, cilia length and prevalence were quantified from maximum intensity projection images. Measurements of cilia length and orientation were calculated using ImageJ as described in our previous paper^21^. Nuclear orientation and aspect ratio were calculated by fitting ellipses to nuclei using ImageJ.

The n values and number of biological replicates are indicated in the figure legends. For experiments using isolated cells, 2–4 random fields of view were imaged per well, resulting in 60–180 cells measured per condition depending on number of biological replicates. In isolated cells the number of cilia was counted for each field and cell number was based on the number of nuclei. The percentage of cilia present in each field was calculated and the data assessed for normality with the Shapiro–Wilk test prior to significance testing with either two tailed unpaired unequal variance Student’s t-tests, 1-factor Anova or 2-factor Anova with post hoc testing using Tukey-Kramer or Tukey-HSD as appropriate and indicated in the corresponding figure legends.

For experiments of cilia expression *in situ* within rat tail tendon, six fascicles were used per condition with 1–3 FM and IFM fields of view for each fascicle yielding 90 ± 30 cells imaged per condition. For comparison with isolated cells fascicles were taken from 3 rats whilst for cyclic loading studies all fascicles were derived from the same animal. Throughout the study cilia length data were pooled, assessed for normality with the Shapiro–Wilk test and then tested for significance with either two tailed unpaired unequal variance Student’s t-tests, 1-factor Anova or 2-factor Anova as appropriate and indicated in the corresponding figure legends. Cilia prevalence for tissue was calculated as the percentage of cells with a cilium of at least 1 μm in length. This threshold was necessary due to difficulty in correctly identifying cilia below 1 μm in tissue. For cilia in tissue total cilia number across all fields was aggregated due to the relatively small number of cells per field, and significance between groups was tested with chi-squared.

Data are available from the corresponding author upon request.

## Results

### Isolation of tenocytes from tendon results in primary cilia elongation and loss of orientation

**Figure 1:**
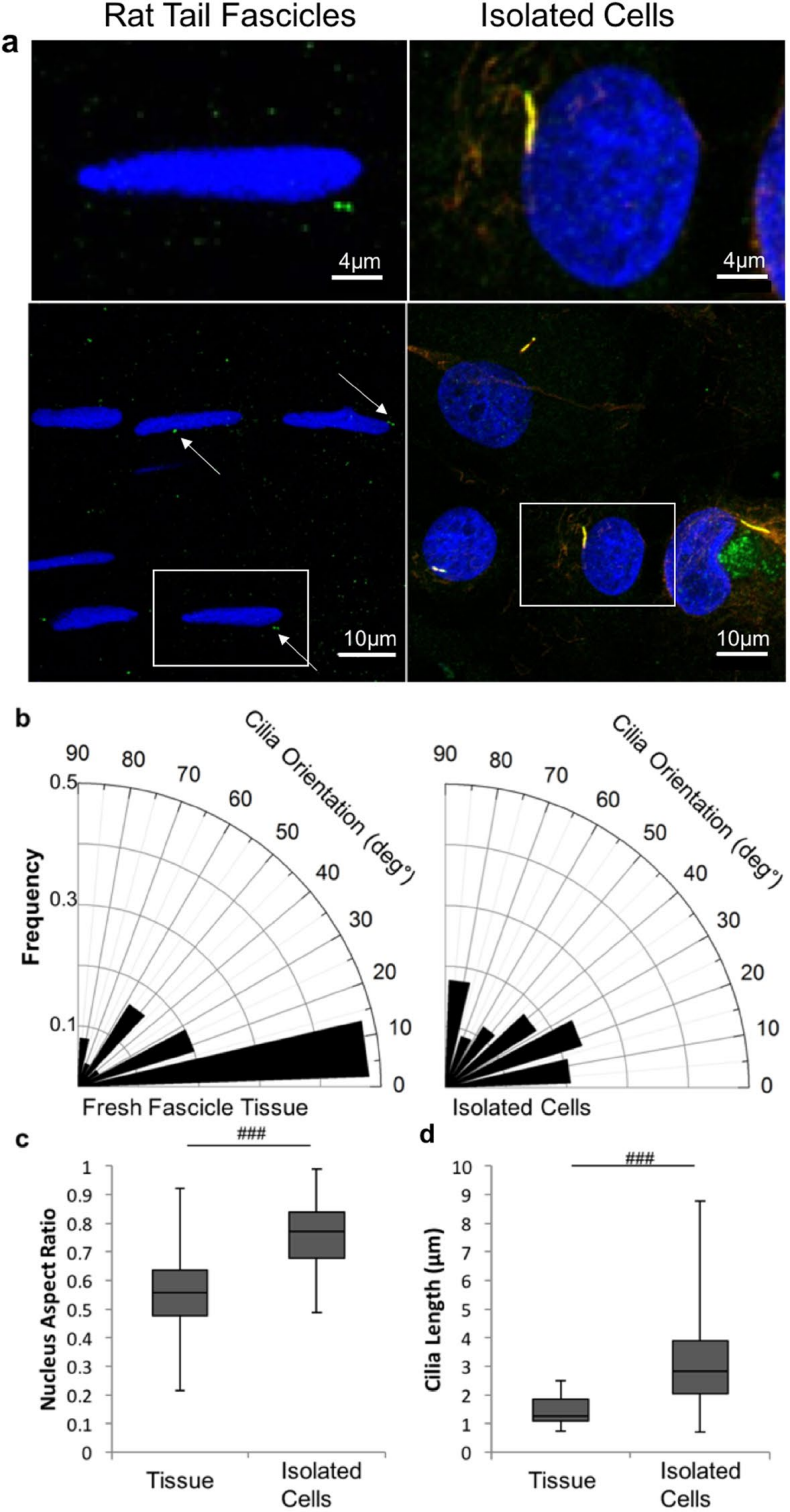
Isolated tenocytes express elongated cilia without any predominate orientation in contrast to that seen in tendon. (**a**) Representative confocal images showing tenocyte primary cilia and nuclei *in situ* within rat tail tendon fascicles (left) and in isolated human cells cultured in monolayer (right) at 2 different scales. The cell nuclei are labelled with Dapi (blue) and cilia are immunolabelled for acetylated α-tubulin (red). In addition, cilia in isolated tenocytes have been immunolabelled for the ciliary membrane protein ARL13b (green). Scale bars represent 10 μm. (**b**) Rose plots showing that the orientation of tenocyte primary cilia is parallel to the long axis of the corresponding nucleus *in situ* within tendon tissue (left) but that there is a lack of any predominant relative orientation in isolated tenocytes (right). (**c**) Box and whisker plot showing nuclear aspect ratio indicating that nuclei in isolated human tenocytes are significantly rounder than those *in situ* in rat tail tendon fascicles. (**d**) Box and whisker plot showing longer primary cilia in isolated human tenocytes compared to *in situ* in rat tail tendon fascicles. N = 120 ± 20 cells and 60 ± 20 cilia per group. For tendon fascicles cilia were imaged from fascicles from 3 rats. For isolated cells images were from 3 donors. All statistically significant differences based on 2 tailed unpaired student’s t-tests are indicated with p < 0.001 represented by ^###^.

Figure 1a shows representative confocal microscopy images of tenocytes and associated primary cilia *in situ* within the fascicular matrix (Left) and following isolation and culture in monolayer (Right). Tenocytes within the tendon had elliptical nuclei with a median aspect ratio of approximately 0.6 (Fig. 1a,c). The primary cilia were orientated in the same direction as the long axis of the nuclei which also reflects the local collagen orientation (Fig. 1a,b). By contrast the nuclei of isolated tenocytes displayed a more rounded morphology as shown by an aspect ratio of approximately 0.8, the difference being statistically significant (p < 0.001, Fig. 1c), and the cilia also displayed no preferential orientation (Fig. 1b). Primary cilia expressed *in situ* within the fascicular matrix were significantly shorter than those in isolated tenocytes in monolayer (Fig. 1a,d). Within the tendon, cilia had a maximum length of approximately 2 μm and a median length of 1.3 µm whilst in isolated tenocytes the maximum length seen was approximately 9 μm with a median length of 3.3 μm (p < 0.001, Fig. 1d).

### Cyclic mechanical loading causes rapid but reversible primary cilia disassembly in isolated tenocytes

**Figure 2:**
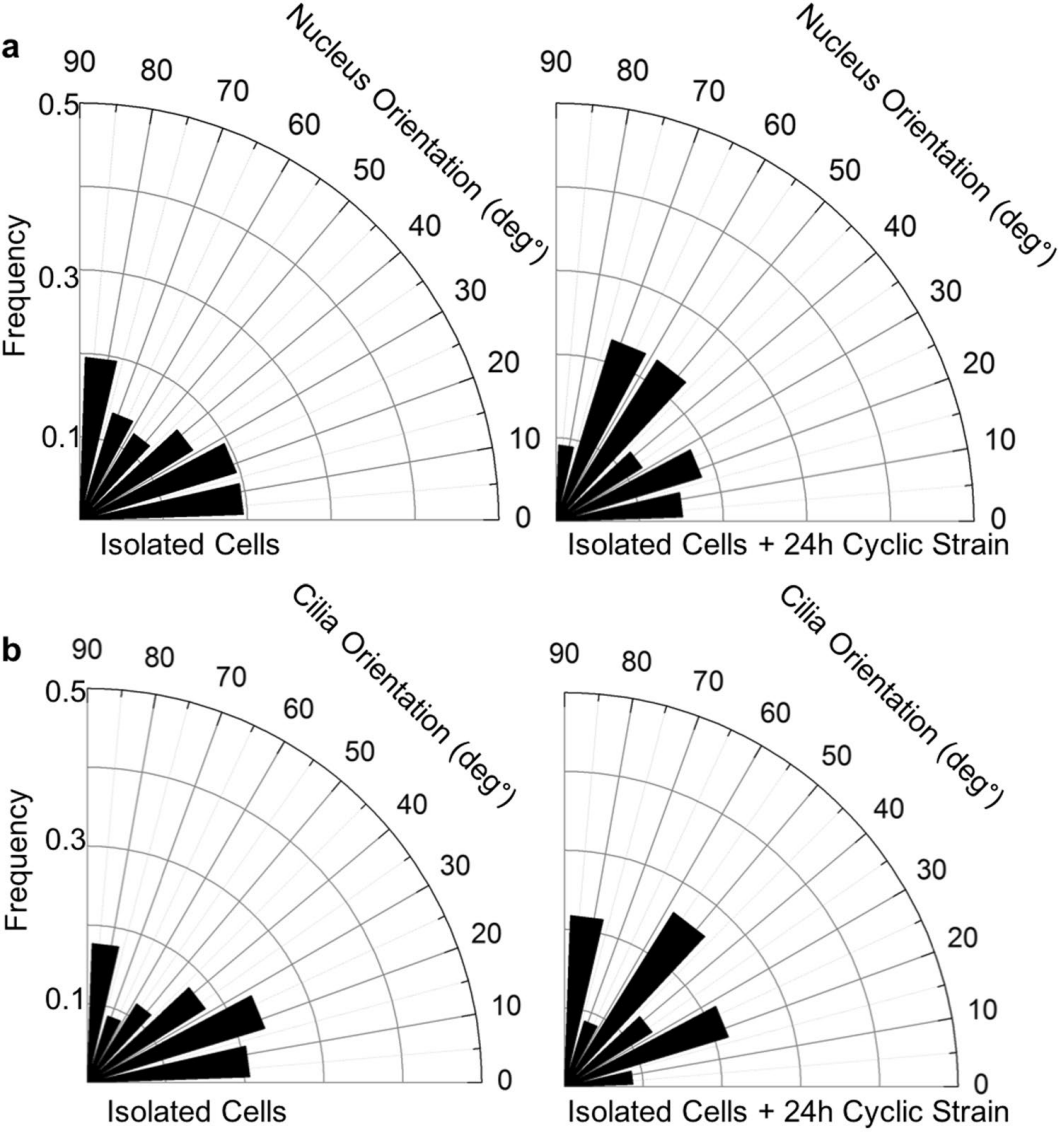
Cyclic tensile strain (24 hours 3%, 1 Hz) does not cause cilia or nucleus reorientation in isolated human tenocytes. (**a**) Rose plots showing lack of any predominant nuclear orientation before and after loading. (**b**) Rose plots showing lack of any predominant cilia orientation relative to nuclear orientation before and after loading. N = 120 ± 20 cells and 60 ± 20 cilia per group, from 3 donors.

**Figure 3:**
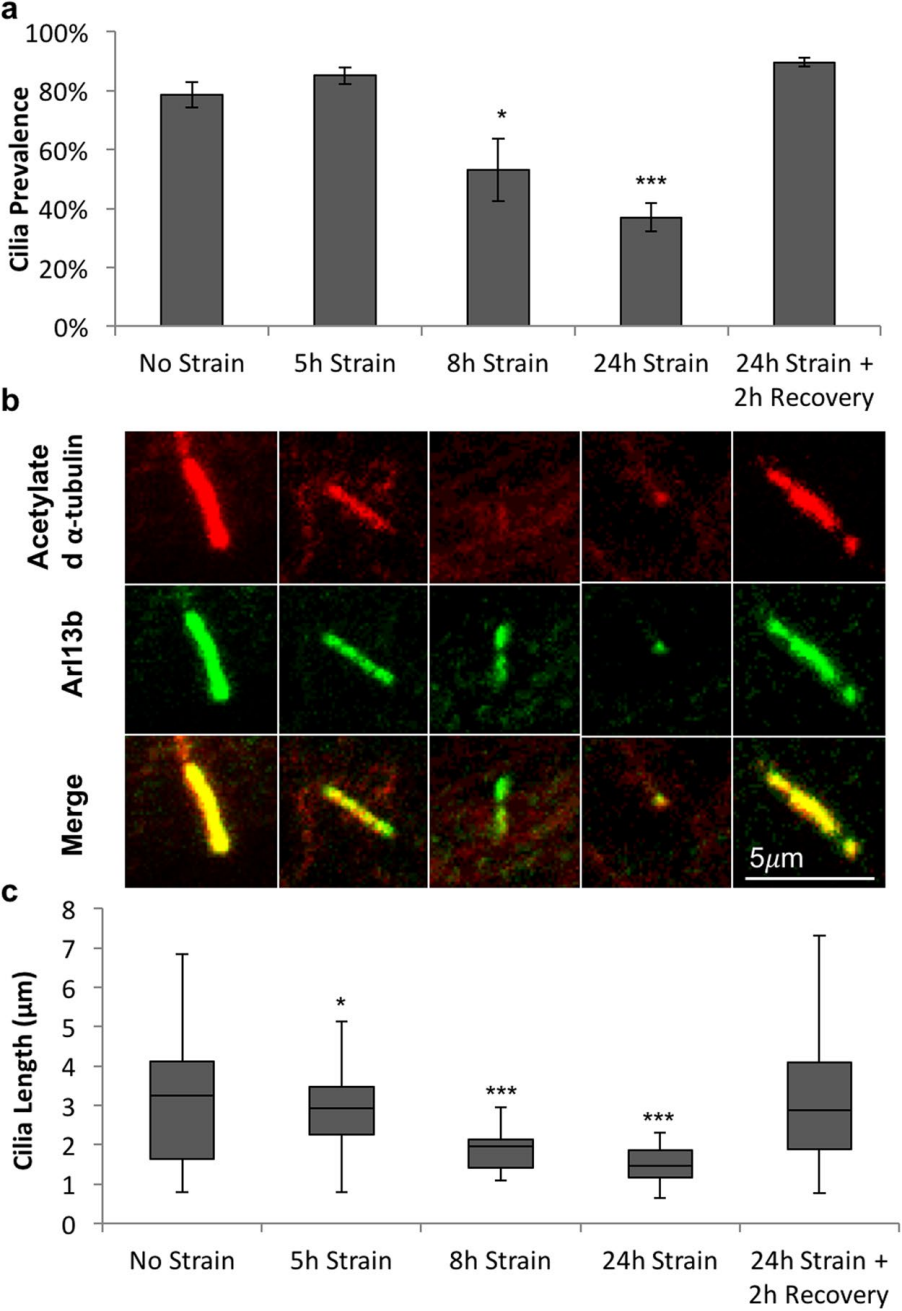
Cyclic tensile strain of isolated human tenocytes leads to progressive disassembly of primary cilia over time which is reversible on strain removal. (**a**) Time course showing the effects of cyclic tensile strain (3%, 1 Hz) on cilia prevalence measured after 5, 8 and 24 hrs of loading and following a 2 hr recovery period after 24 hrs of loading. Values represent mean prevalence with error bars showing standard error (n = 6 fields of view per condition). (**b**) Representative confocal images of primary cilia in unloaded cells and loaded cells. Cilia have been labelled with anti-acetylated α-tubulin (red) and anti-ARL13b (green). Scale bar represents 5 μm. (**c**) Box and whisker plots showing corresponding cilia length. N = 160 ± 20 cells and 80 ± 40 cilia per condition from across 3 donors. Data were analysed with 1-factor Anova. Data was significant at p < 0.001. Post hoc testing was performed using Tukey-Kramer. Statistically significant differences relative to unstrained control are indicated at p < 0.05 (*), p < 0.01 (**) and p < 0.001 (***).

The application of 24 hrs cyclic mechanical loading (0–3%, 1 Hz) to isolated tenocytes did not have any apparent effect on the random orientation of either the cell nuclei (Fig. 2a) or the primary cilia (Fig. 2b). However, loading did induce progressive cilia disassembly. There were no significant differences observed after 5 hrs of loading, but by 8 hrs there were significant reductions in both cilia prevalence (p < 0.05, Fig. 3a) and cilia length (p < 0.05, Fig. 3b,c). There was further cilia disassembly after 24 hrs with only 35% of tenocytes expressing cilia compared to 80% of unloaded control cells (p < 0.001, Fig. 3a). Similarly, for those cells that did still express a cilium, there was a significant reduction in length of more than 50% with median values of 1.5 μm (p < 0.001, Fig. 3b,c). Despite this dramatic cilia disassembly, the removal of mechanical loading led to a complete recovery of both cilia prevalence (Fig. 3a) and length (Fig. 3b) within 2 hrs, such that neither parameter was significantly different from values in unloaded controls.

### Cyclic mechanical loading causes cilia disassembly in the fascicular but not interfascicular matrix regions of the tendon

**Figure 4:**
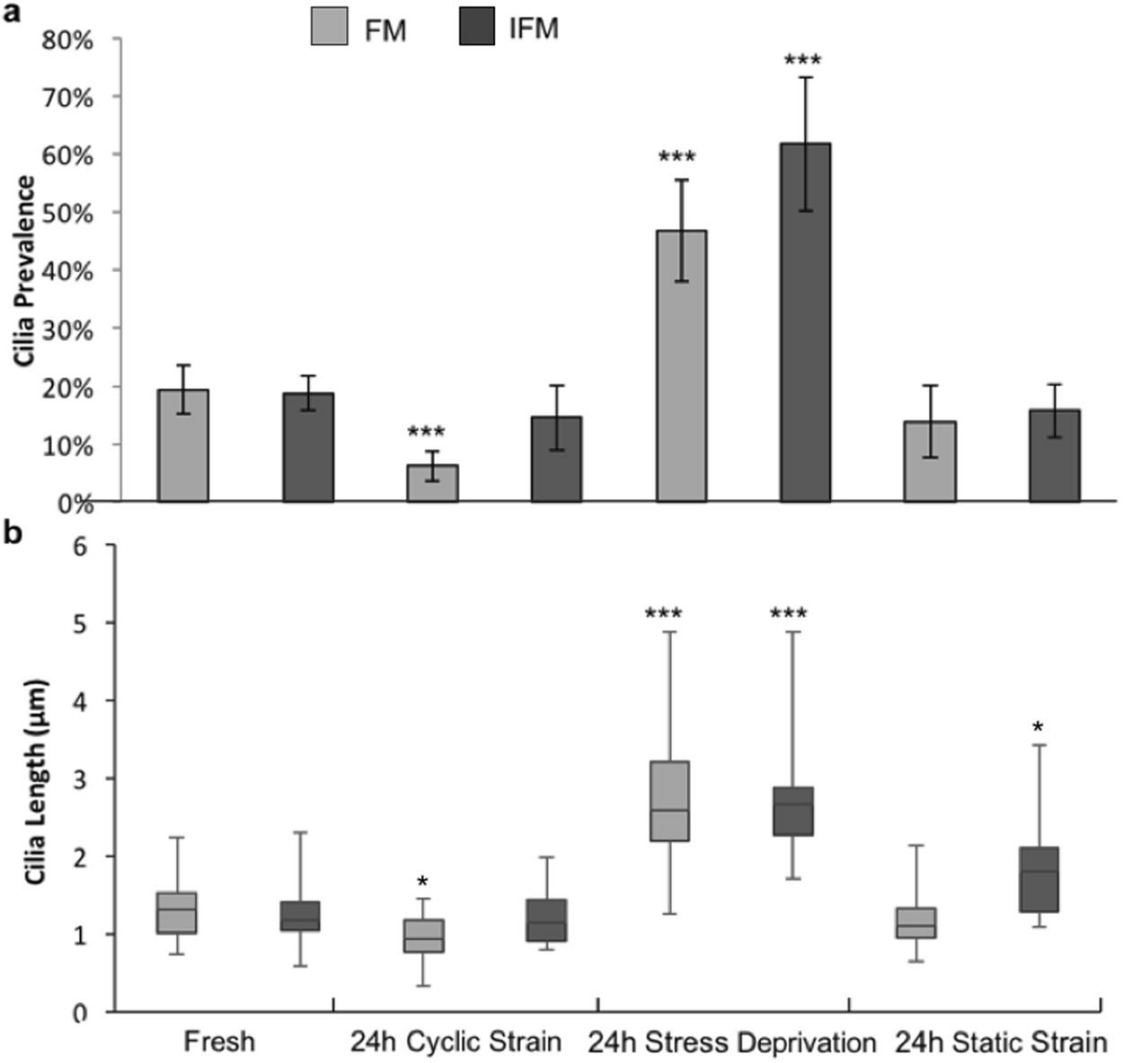
Cyclic tensile strain leads to cilia disassembly *in situ* within tendon tissue. Primary cilia (**a**) prevalence and (**b**) length in the FM and IFM region of fresh rat tail tendon fascicles and following 24 hours of either cyclic tensile strain (4%, 1 Hz); stress deprivation or static tensile strain (4%). Prevalence values represent mean with error bars indicating Poisson standard error. N = 10–50 cilia per condition from 90 ± 30 observed cells from fascicles from 1 rat. Box and whisker plot show cilia length. Significance for prevalence data was calculated using chi-squared tests. Length data were analysed with 2-factor Anova. Significance at the 0.001 level was found for both tissue region and treatment type but interactions were not found to be significant at the 0.05 level. Post hot testing was done using Tukey-Kramer. Statistically significant differences are indicated between FM and IFM and relative to corresponding values for fresh fascicles, p < 0.05 (*) and p < 0.001 (***).

We next examined whether mechanically induced cilia disassembly was also present following cyclic mechanical loading of intact tendon fascicles. These studies examined the effect of loading on cilia within both the fascicular matrix (FM) and interfascicular matrix (IFM). Similar to behaviour in isolated tenocytes, 24 hr cyclic loading (0–4%, 1 Hz) led to significant reductions in both cilia prevalence (p < 0.001, Fig. 4a) and cilia length (p < 0.001, Fig. 4b) for tenocytes within the fascicular matrix compared to fresh tendon sample. By contrast, no significant reduction in length or prevalence were observed for cells in the interfascicular matrix (Fig. 4a,b). In the stress deprived controls, cilia prevalence increased by more than 2-fold compared to values in fresh tendon fascicles and interfascicular matrix (p < 0.001, Fig. 4a). Cilia length also increased in both the FM and IFM regions with median lengths of 2.5 μm and 2.6 μm respectively (p < 0.001, Fig. 4b). Notably, the values of cilia length in stress deprived rat tail tendon fascicles are similar to those for isolated human tenocytes in unloaded conditions. In contrast to the response to cyclic loading, the application of static strain (4%, 24 hrs) induced no difference in cilia prevalence compared to fresh tendon in either the FM or IFM regions (Fig. 4a), but did lead to a small but significant increase in the length of cilia in the IFM region (p < 0.001, Fig. 4b), as was seen in our previous paper^21^.

### Mechanically induced cilia disassembly in tenocytes is mediated by TGFβ and HDAC6, independent of actin changes

**Figure 5:**
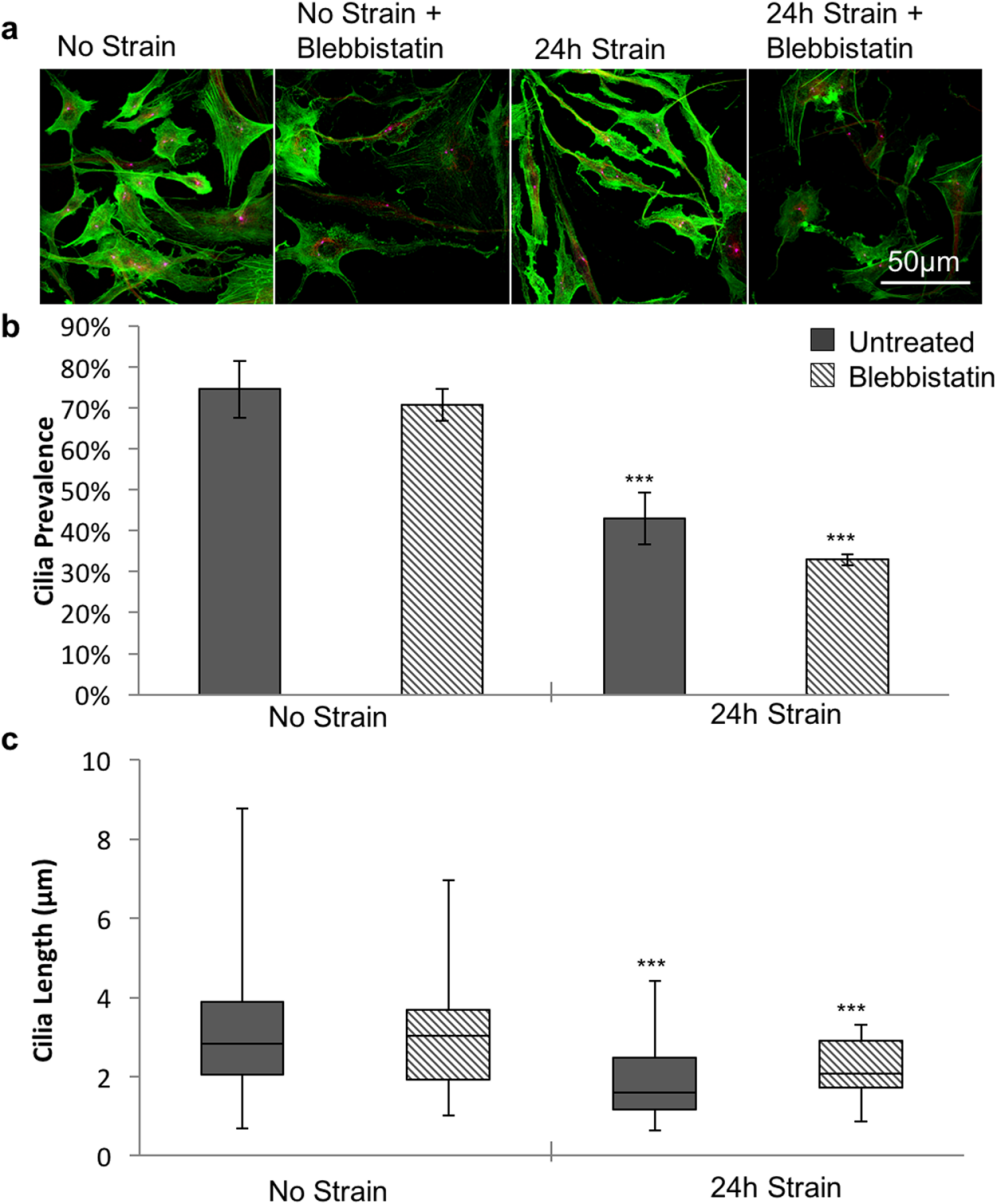
Cilia disassembly induced by cyclic strain is not caused by changes in actin tension. (**a**) Representative confocal images showing F-actin in unstrained isolated human tenocytes and following a 24 hour period of cyclic tensile strain (3%, 1 Hz) with and without treatment with 10μM Blebbistatin. Actin labelled with Alexa-Phalloidin. Scale bars represent 50 μm. (**b**) Corresponding cilia prevalence (values represent mean with error bars indicating standard error for n = 4 fields for each condition). (**c**) Box and whisker plots showing corresponding cilia length for each condition (N = 40 ± 20 cilia per condition from 1 donor). Data were analysed with 2-factor Anova for both length and prevalence. Significance was observed at the 0.001 level between strain and no strain. However, no significance was observed between blebbistatin and no blebbistatin nor was any significance noted between the interactions. Post hoc testing was performed using Tukey-Kramer. In all cases significance is indicated at p < 0.05 (*), p < 0.01 (**) and p < 0.001 (***).

Mechanical loading has been shown to increase actin tension and stress fibres perpendicular to the direction of strain organisation in fibroblasts^26^, whilst further studies show that changes in actin tension can regulate cilia expression in various other cell types^32,33^. To investigate whether reorganisation of the actin cytoskeleton was responsible for the mechanically induced cilia disassembly, we treated cells with blebbistatin to reduce actin tension. Confocal visualisation of F-actin in isolated tenocytes demonstrated that with the loading conditions used in this study (0–3%, 1 Hz, 24 hr), cyclic tensile strain had minimal effect on actin organisation (Fig. 5a). Furthermore, although blebbistatin significantly reduced the presence of actin stress fibres (Fig. 5a), this had no significant effect on cilia prevalence (Fig. 5B) or length (Fig. 5c) in either the unloaded control or cyclic loaded conditions. Together these data indicate that mechanical loading did not induce cilia disassembly via actin reorganisation.

**Figure 6:**
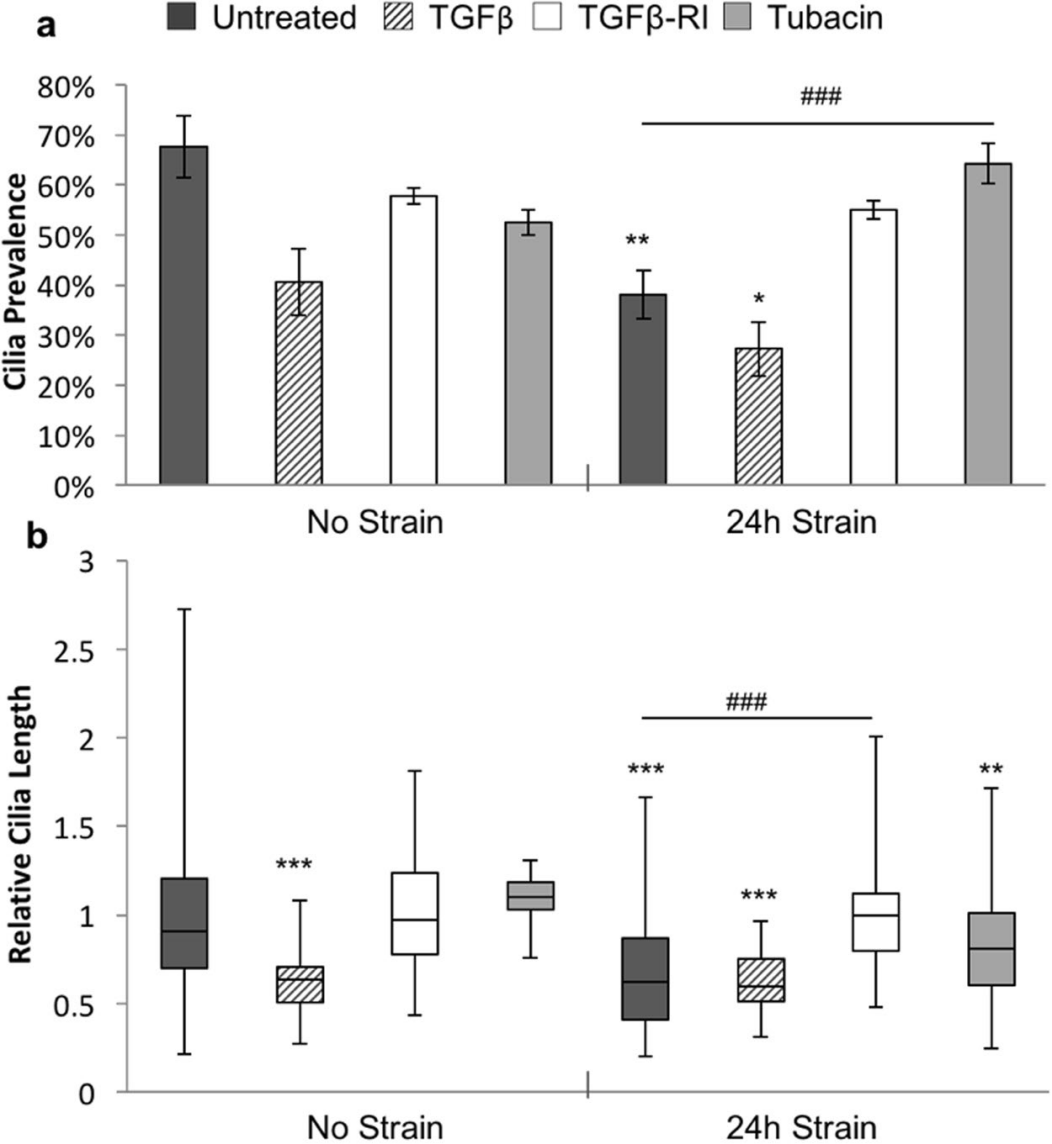
Cilia disassembly induced by cyclic strain is caused by activation of TGFβ receptors and HDAC6. Primary cilia (**a**) prevalence and (**b**) length in unstrained cells and cyclic strained cells (24 hours, 3%, 1 Hz) for cells treated with either TGFβ; TGFβ receptor inhibitor (TGFβ-RI) or tubacin. For comparison across different experiments cilia length has been normalised to the corresponding unstrained control. Prevalence data represent mean values with error bars indicate standard error for n = 8 ± 4 fields of view. Corresponding relative lengths are shown as box and whisker plots for N = 120 ± 60 cilia. Images taken from cells from 3 donors for untreated cells, TGFβ-RI treated cells and tubacin treated cells, images were taken from a single donor for TGFβ treated cells. Data analysed using 2-factor Anova. Groups and interactions were significant at p < 0.001 for both length and prevalence. Post-hoc testing was performed using Tukey-Kramer. Statistically significant differences are indicated relative to unstrained control at p < 0.05 (*), p < 0.01(**), p < 0.001 (***) and between groups p < 0.05 (^#^), p < 0.01(^##^), p < 0.001 (^###^).

We next investigated the role of TGFβ, which has been shown to mediate mechanosignalling in tendon^7^. Mechanically loaded (0–3%, 1 Hz, 24 hrs) and unloaded cells were treated with TGFβ or the receptor inhibitor (SB 431542 hydrate TGFβ-RI) prior to analysis of cilia expression. In addition, previous studies have demonstrated that mechanically induced cilia disassembly and shortening in chondrocytes is mediated by the histone deactylase, HDAC6^24,28,29^. Therefore separate groups of loaded and unloaded cells were treated with the HDAC6 inhibitor, tubacin, and results compared to untreated controls. Treatment with TGFβ led to a significant reduction in cilia length (p < 0.001, Fig. 6b), in unloaded cells. Indeed, the effects of TGFβ were similar to those induced by mechanical loading with no significant differences between the two groups and the addition of loading to TGFβ treated cells did not further enhance cilia disassembly in terms of cilia prevalence (p = 0.16) or length (p = 0.22). In unloaded cells, TGFβ-RI had no significant effect on cilia expression (p > 0.05). However in loaded cells, inhibition of the TGFβ receptor prevented mechanically induced cilia disassembly such that there were no significant differences between loaded + TGFβ-RI and unloaded control cells. These results demonstrate that mechanically induced cilia disassembly in tenocytes is mediated by activation of TGFβ receptors.

In the absence of strain, inhibition of HDAC6 with tubacin had no significant effect on cilia prevalence or length (Fig. 6). However, in loaded cells, tubacin rescued the mechanically induced reduction in cilia disassembly, such that values were significantly different from untreated, loaded conditions (p < 0.001). Cilia prevalence was completely restored to levels in unstrained, untreated cells, however, cilia length remained slightly shorter.

## Discussion

Previous studies from our group and others have described the response of tenocyte primary cilia to mechanical loading of tendon tissue^18,19,21^. This study is the first to examine primary cilia in isolated tenocytes and the effect of mechanical loading as well as the underpinning mechanism. By studying isolated cells it is possible to identify the direct effect of mechanical loading in isolation from factors associated with loading or remodelling of the extracellular matrix. Isolated tenocytes from human tendon, cultured in monolayer in the absence of mechanical loading exhibited primary cilia that were significantly longer than those in intact rat tail tissue (Fig. 1). Interestingly the cilia set length in unloaded isolated tenocytes is similar to that previously reported in stress deprived tendon^18,21^. Although species-to-species variation cannot be ruled out, these results suggest that cilia elongation in response to stress deprivation *in situ* is directly due to the absence of loading rather than any indirect effect caused by remodelling of the surrounding matrix.

Primary cilia expressed by isolated tenocytes had no predominant orientation compared with cilia within the tendon fascicular matrix (Fig. 1). Furthermore, no obvious reorientation of either cilia or nuclei was observed in isolated cells after 24 hours of cyclic tensile strain. This is in contrast to other cell types where nuclei have been observed to reorient perpendicularly to the axis of applied strain depending on confluence, loading frequency and duration^34^. The lack of orientation in isolated cells suggests that the orientation observed in tendon fascicular matrix (FM) is due to constraint by the collagen fibres of the fascicle which are orientated parallel to the applied strain, rather than the strain itself. This may also explain the lack of orientation reported in the interfasicular matrix (IFM) where the collagen fibres are more randomly orientated relative to the axis of loading.

Cyclic loading of isolated tenocytes induced a marked reduction in cilia prevalence and set length with increasing loading duration. This response to loading was replicated in the fasicular matrix (FM) region of tendon fascicles after 24 hours of cyclic loading (4% 1 Hz). No significant reduction in prevalence or length was observed in the interfascicular matrix (IFM) region, which may be due to lower strains in this region of the tendon compared to the fascicle itself ^35^.

We next examined the mechanism for mechanical regulation of cilia expression. Previous studies have reported that loading modulates actin organisation in other cell types^26^ and that increased actin tension inhibits cilia elongation^24^. We therefore treated cells with Blebbistatin to reduce actin tension as indicated by confocal imaging of cells labelled with Alexa-phalloidin. Despite this alteration in actin organisation, Blebbistatin had no significant effect on cilia prevalence or length (Fig. 5). Furthermore, Alexa-phalloidin imaging demonstrated that cyclic strain had no obvious effect on actin organisation. These results therefore suggest that alterations in actin are not involved in the mechanism of mechanical regulation of cilia expression.

We next examined an alternative mechanism involving TGFβ, which has previously been shown to be activated by mechanical loading of tenocytes^7^. Previous studies have reported that TGFβ reduces cilia expression in mesenchymal stem cells and osteoblasts^28,36^. We found that application of TGFβ_1_ to unloaded isolated tenocytes induced a significant reduction in both cilia prevalence and set length, similar to that observed with loading. Furthermore, treatment of cyclically loaded cells with TGFβ receptor inhibitor (SB 431542) blocked mechanically-induced changes in cilia expression without affecting unstrained cells. It has previously been shown that mechanical loading of chondrocytes induces cilia disassembly and shortening via the activation of histone deacetylase 6 (HDAC6) which is enriched on the cilium^29^. Here we show that treatment of tenocytes with the HDAC6 inhibitor (Tubacin) prevented mechanically-induced reductions in cilia prevalence and length. Together, these results demonstrate that mechanical regulation of cilia expression in tenocytes is mediated by activation of TGFβ_1_ receptors and HDAC6.

**Figure 7:**
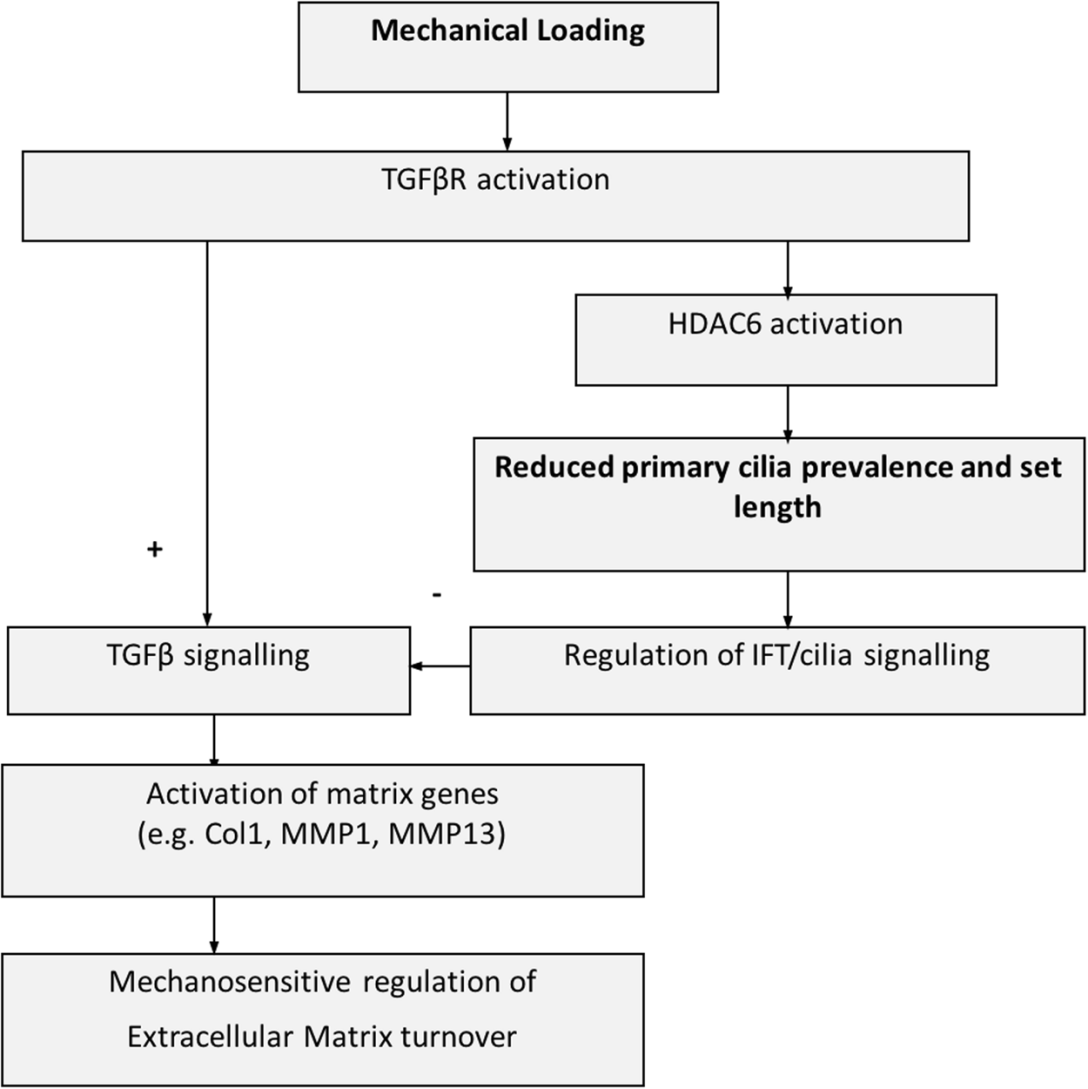
Schematic diagram showing proposed pathway through which cyclic mechanical loading regulates primary cilia expression to control tendon matrix turnover. Mechanical loading disrupts primary cilia expression via TGFβR and HDAC6 activation. This in turn may modulate cilia signalling, including suppression of TGFβ signalling as part of a negative feedback mechanism regulating tendon matrix turnover in response to mechanical loading.

The relationship between cilia length and function is unclear and hence the consequences of mechanically-induced tenocyte cilia disassembly are unknown. Elongation of osteocyte primary cilia using fenoldopam or lithium has been shown to be associated with increased ciliary mechanosensitivity^37^. In chondrocytes, mechanically-induced cilia disassembly inhibits hedgehog signalling^29,38^. Previous studies have also shown that cilia set length regulates injection of intraflagellar transport (IFT) cargoes as part of the balance point model first proposed by Marshall^39^. Thus mechanical loading of tendon may effect a variety of cilia signalling pathways by TGFβ_1_-mediated regulation of tenocyte cilia length and subsequent control of IFT. Interestingly, studies in mesenchymal stem cells show that TGFβ receptors localise to the ciliary base, and that TGFβ signalling is attenuated in the absence of cilia^15^. Thus loading-induced changes in cilia expression may regulate TGFβ signalling as part of a negative feedback mechanism. This mechanism may be protective to ensure that TGFβ_1_-activated genes, such as MMP1, MMP13 and Col1A1, are not continually upregulated if loading continues for a long period of time. This could explain why cyclic loading induces transient activation of these genes. A schematic diagram demonstrating this hypothesis is shown in Fig. 7.

## Conclusion

This study is the first to describe primary cilia expression and the effect of mechanical loading in isolated tenocytes. We reveal that loading induces time dependent, recoverable loss of primary cilia and a reduction in cilia set length. We show that this response does not involve changes in actin organisation, but is mediated by activation of TGFβ receptors leading to HDAC6-dependent cilia disassembly. We suggest that this may represent a novel feedback mechanism controlling TGFβ and other signalling pathways which regulate tendon extracellular matrix turnover. Manipulation of this pathway may provide novel therapeutic approaches for regulation of tendinopathy associated with excessive or prolonged mechanical loading.
